# Total sitting time and risk of myocardial infarction, coronary heart disease and all-cause mortality in a prospective cohort of Danish adults

**DOI:** 10.1186/1479-5868-11-13

**Published:** 2014-02-05

**Authors:** Christina Bjørk Petersen, Adrian Bauman, Morten Grønbæk, Jørn Wulff Helge, Lau Caspar Thygesen, Janne S Tolstrup

**Affiliations:** 1 National Institute of Public Health, University of Southern Denmark, Øster Farimagsgade 5A, Copenhagen 1353, Denmark; 2 School of Public Health, University of Sydney, Sydney NSW 2006, Australia; 3 Center of Healthy Ageing, Department of Biomedical Sciences, University of Copenhagen, Blegdamsvej 3, Copenhagen 2200, Denmark

**Keywords:** Sitting time, Physical activity, Longitudinal studies, Myocardial infarction, Coronary disease, Mortality, Denmark

## Abstract

**Background:**

Evidence suggests that sitting time is adversely associated with health risks. However, previous epidemiological studies have mainly addressed mortality whereas little is known of the risk of coronary heart disease. This study aimed to investigate total sitting time and risk of myocardial infarction, coronary heart disease incidence and all-cause mortality.

**Methods:**

In the Danish Health Examination Survey (DANHES) conducted in 2007-2008 we tested the hypothesis that a higher amount of daily total sitting time is associated with greater risk of myocardial infarction, coronary heart disease and all-cause mortality. The study population consisted of 71,363 men and women aged 18-99 years without coronary heart disease. Participants were followed for myocardial infarction, coronary heart disease and mortality in national registers to August 10, 2012. Cox regression analyses were performed with adjustment for potential confounders and multiple imputation for missing values.

**Results:**

During a mean follow-up period of 5.4 years 358 incident cases of myocardial infarction, 1,446 of coronary heart disease, and 1,074 deaths from all causes were registered. The hazard ratios associated with 10 or more hours of daily sitting compared to less than 6 hours were 1.38 (95% CI: 1.01, 1.88) for myocardial infarction, 1.07 (95% CI: 0.91, 1.27) for coronary heart disease and 1.31 (95% CI: 1.09, 1.57). Compared to sitting less than 6 hours per day and being physically active in leisure time, the hazard ratios of sitting more than 10 hours per day and also being physically inactive in leisure time were 1.80 (95% CI: 1.15, 2.82) for myocardial infarction, 1.42 (95% CI: 1.11, 1.81) for coronary heart disease, and 2.29 (95% CI: 1.82, 2.89) for all-cause mortality.

**Conclusions:**

The results suggest that a higher amount of daily total sitting time is associated with all-cause mortality, particularly among inactive adults. In relation to coronary heart, disease results were less clear. This paper adds new evidence to the limited data on the evidence of sitting time and cardiovascular disease and mortality.

## Background

A rapidly expanding body of evidence suggests that high amounts of daily sitting time are associated with increased risk of adverse health outcomes in adults, even among individuals who are physically active in leisure time [1-3]. Studies have found that working adults are sitting down for about one third to one half of their workday [4-7]. In addition to sitting at work, adults also spent hours sitting in leisure-time and during transportation. Thus, prolonged sitting time constitutes a public health concern. Prospective studies have shown that sedentary behavior is associated with all-cause mortality and cardiovascular disease mortality [8-15]. Few studies have specifically examined the association between sedentary behavior and incident coronary heart disease (CHD) [16-20]. In three of such studies sitting time was measured as screen time or time spend watching television [16,19,20]. However, other domains also contribute to daily sitting time as for example sitting during transportation and at work. Results from two studies that focused on total sitting time, both included women only and showed conflicting results [17,18]. Manson *et al*. found an increased risk of CHD among women with a daily sitting time of at least 16 hours compared to less than four hours [18]. In contrast, Herber-Gast *et al*. found no significant association between sitting time and CHD incidence [17]. Therefore, evidence on the association between sitting time and CHD incidence is sparse and inconsistent. For this reason, and because of the high public health relevance, the aim of this study was to investigate total sitting time and risk of myocardial infarction (MI), CHD and all-cause mortality in a large prospective cohort of both men and women.

## Material and methods

### Study population

The study was based on the Danish Health Examination Survey (DANHES) which was conducted in 13 municipalities in Denmark in 2007 and 2008. The DANHES study design is described in detail elsewhere [21]. In each municipality, all citizens aged 18 or more were invited to participate. The study was conducted over a period of one month in each municipality. A total of 538,163 persons were invited to the DANHES study and 76,484 persons participated (response rate: 14%). Women in general, but especially women in the age group 45–64 years, were over-represented in DANHES compared with the Danish population, whereas younger men, older women, and those with lower education or income or who were not married were underrepresented.

The self-administrated DAHNES questionnaire included questions regarding general health, morbidity, well-being, lifestyle, and social relations. In 12 municipalities, an internet-based version of the questionnaire was used. In one municipality, a paper questionnaire was used. Besides the questionnaire, a random subsample was invited to participate in a clinical health examination including blood samples (n = 180,103). Of these 18,065 participated (participation rate: 10%). As for the questionnaire survey, women in general, but especially women in the age group 45–64 years, were overrepresented, whereas younger men and women and those with lower education were underrepresented. Participants provided informed consent and the protocol was reviewed by the Scientific Ethical Committee B for the Capital Region of Denmark (H-B-2007-050).

Participants with pre-existing CHD were identified by self-reported questionnaire at baseline (n = 3132) and by linkage to the Danish National Patient Register (n = 1725). Participants were asked to rate their health in 5 pre-defined categories (very good, good, fair, poor and very poor). To minimize the risk of including preclinical cases, we excluded individuals with very poor self-reported health at baseline (n = 258). Finally, 6 individuals were excluded due to errors in the registers of the time of death leaving 71,363 individuals for the analysis. For the analysis with plasma cholesterol, triglycerides, waist circumference and body mass index (BMI) as outcomes, only individuals participating in the health examination were included (n = 16,049).

### Assessments

#### MI, CHD and mortality

Information on MI and CHD incidence was obtained from the Danish National Patient Register where all hospitalizations in Denmark are registered. MI was defined according to the International Classification of Diseases (ICD) by the ICD-8 code 410 and the ICD-10 codes I21–I22 (acute MI and subsequent MI). CHD was defined by ICD-8 codes 410–414 and ICD-10 codes I20–I25 (angina pectoris, acute MI, subsequent MI, certain current complications following acute MI, other acute CHD and chronic CHD). Incident MI and CHD included both fatal and non-fatal cases. Data on all-cause mortality were obtained from the Danish Civil Registration System.

#### Triglycerides, total cholesterol, waist circumference and BMI

Non-fasting venous blood samples were collected for examination of P-cholesterol and P-triglyceride at the clinical health examination conducted at baseline. The blood sample collection was carried out by the Department of Clinical Biochemistry, KB 3-01-1, Rigshospitalet. Non-fasting venous blood samples were collected from fossa cubiti after light stasis into a total of five tubes – three lithium-heparin tubes (of 3 ml) and two K3-EDTA tubes (of 2 ml). Blood samples from heparin tubes were kept at room temperature less than two hours before being centrifuged. All tubes were stored at the place of data collection at 5°C and were transported weekly to the Department of Clinical Biochemistry. The blood samples were analyzed based on guidelines from the Department of Clinical Biochemistry 0–8 days from arrival at the laboratory. The waist circumference (cm) was measured with body tape at a level midway between the lower rib margin and the iliac crest. BMI (kg/m^2^) was calculated from height measured without shoes and body weight measure by a Body Composition Analyzer (type BC-418 MA, Tanita Corp., Tokyo, Japan).

#### Sitting time

Daily total sitting time was measured by a Danish version of the long International Physical Activity Questionnaire (IPAQ). IPAQ is known to be a valid and reliable instrument for assessing physical activity and sitting time in a European setting such as Denmark [22]. It consists of 31 items that collect information on physical activity in the four domains: work, transport, housework/gardening and leisure time, and time spent sitting. Sitting time was assessed by the following question: “During the last 7 days, how much time did you usually spend sitting during work and leisure time on: a) a weekday? and b) a weekend day? The sitting question was prefaced by asking participants to consider multiple life domains of sitting excluding time spent sitting during transport, which was captured in another item of the IPAQ questionnaire. Average total sitting time (minutes per day) was calculated as the sum of weekday sitting minutes*5 and weekend day sitting minutes*2, and divided by 7. Time spent travelling in a motor vehicle was also added (minutes per day). Lastly the sum was converted to hours per day.

#### Covariates

Leisure time physical activity was assessed as the average physical activity level during the last year, graded in four levels based on the original item as proposed by Saltin and Grimby [23] with minor modifications: (1) vigorous (strenuous activities usually involving competition or endurance training performed regularly or several times a week); (2) moderate (exercise, endurance training, or heavy gardening for at least 4 h a week); (3) light (walking, bicycling (including cycling/walking during transportation), or other light activities for a minimum of 4 h a week); (4) inactive (reading, TV-watching, or other sedentary activities). For joint analysis with sitting time, leisure time physical activity was dichotomized: (1) Level 1, 2 and 3 combined was considered physically active and level 4 was considered physically inactive. In addition, total physical activity and leisure-time physical activity were measured by IPAQ. Participants reporting days but not time of physical activity were treated as missing. In each domain, minutes spent on physical activity at moderate and vigorous intensity and walking for more than 180 min per day were truncated to 180 min. Durations between 0 and 10 min was accepted even though the questionnaire was restricted to activity of at least 10-min duration. Total time spent in each domain were calculated and scored by MET values according to guidelines from the IPAQ group [24]. For joint analysis with sitting time, physical activity was dichotomized: 1) below or equal the 25th percentile, 2) above the 25th percentile.

Additional questionnaire variables included in the analyses were: age, education (<10 years, 10–12 years, 13-14 years and 15+ years), smoking (never-smoker, ex-smoker, occasional smoker, daily smoker [1–15 g of tobacco/day], and heavy smoker [>15 g of tobacco/day]), alcohol consumption (0 drinks/week, 1- < 7 drinks/week, 7- < 14 drinks/week, 14– < 21 drinks/week, and 21+ drinks/week), self-reported diabetes measured by a question of “have you ever been told by a doctor that you have diabetes?” (yes, no), self-reported hypertension (yes current/yes previously, no), and BMI (<25 kg/m^2^, 25– < 30 kg/m^2^, and >30 kg/m^2^).

### Statistical analyses

Descriptive analysis involved univariate comparisons of continuous and binary variables using ANOVA and *χ*2 tests. Around 18% of the values of total sitting time were missing (17% for transportation, 8% for sitting during weekends and 5% for sitting during weekdays). To account for missing values we performed multiple imputations by chained equations (m = 20 imputations) [25]. The imputation procedure included the outcome of interest (CHD, MI or all-cause mortality) and the main exposure (sitting time during transport, on weekdays and at weekends) as well as other covariates (age, sex, educational level, physical activity level in leisure time, smoking habits, BMI, alcohol consumption and hypertension). Analyses using multiple imputations were predetermined as the primary analyses and secondary complete case analyses were conducted (Additional file 1).

Previous studies on sitting time and various health outcomes have indicated a possible threshold for total sitting time between 8 and 11 hours per day. When fitting the model, we examined the linear and non-linear association between sitting time and health outcomes by splines (knots at 8 and 10 hours of sitting time per day). Also, we applied fractional polynomials with linear, squared, cubic, and square-root terms for total sitting time [26]. Based on the above mentioned analyses and prior knowledge, we treated sitting time categorically in the analyses: 1) < 6 hours per day 2) 6- < 10 hours per day 3) ≥10 hours per day. Tests of linear trend were calculated by assigning the median value within each sitting category and treating it continuously in the model.

Cross-sectional associations between sitting time and total cholesterol, triglycerides, BMI and waist circumference were investigated by multiple linear regression models adjusting for potential confounding factors (age, alcohol consumption, smoking habits, and education). Also, analysis with cholesterol and triglycerides were adjusted for BMI and waist circumference. Triglyceride was log-transformed to approximate normal distribution and back transformed in reported results.

The association between sitting time and MI and CHD incidence as well as all-cause mortality was analyzed using Cox proportional hazards models adjusting for potential confounding factors (education, physical activity in leisure time, smoking habits, alcohol consumption, BMI, hypertension and diabetes). In a sub-analysis, results were further adjusted for diet (weekly consumption of fish and daily consumption of fruits, vegetables and fiberrich bread and grains) but omitted in the main analysis due to the crude measure of diet and because it did not alter results. Age of the participants was applied as the underlying time scale to remove the confounding by age. Each individual contributed person-time from return of the questionnaire in 2007/2008 to the date of a first coronary event, date of death or August 10, 2012, whichever came first. We had follow-up information on all participants. Evaluation of the assumption about proportional hazards was done by visual inspections of log-log plots and tested using Schoenfeld residuals. We examined the interplay between total sitting time and physical activity in leisure time by combining categories of sitting time and physical activity in leisure time. To support results, we repeated analysis using total physical activity and leisure-time physical activity as measured by the IPAQ questionnaire. We also examined the sitting time stratified by physical activity in leisure time using nested models with and without interaction terms.

Results did not differ by gender and therefore results are shown combined for men and women with the exception of analysis with total cholesterol, triglycerides, BMI, and waist circumference as outcome. Due to the skewed distribution of participants in the DANHES compared to the general Danish population, calibrated weights were computed based on register information on sex, age, geography, educational level, income, and civil status for all individuals who were invited in the 13 municipalities (both participants and non-participants). Hence, it was to a certain extent possible to statistically allow for the differential non-response by using auxiliary information from Statistics Denmark’s registers.

In sensitivity analyses, we delayed the start of follow-up by two years to minimize the effect of a pre-existing disease. Also, for analyses with all-cause mortality, we excluded participants with diagnosis of cancers and all cardiovascular diseases. We also evaluated the rates of CHD and all-cause mortality according to total sitting time in individuals meeting four criteria of healthy lifestyle: BMI < 25, not smoking, alcohol consumption below 14 drinks/week for women and 21 drinks/week for men, and being at least lightly physically active in leisure time.

Analyses were performed using STATA 12.0 statistical software.

## Results

During the study period from 2007-2008 to 2012, 358 were diagnosed with MI, 1,446 were diagnosed with CHD and 1,074 individuals died. The mean age at baseline was 48 years. The median total sitting time was 6.9 hours per day (25th-75th percentiles = 4.5-8.7). More than half of the participants reported a light physical activity levels in leisure time and 14% were inactive.

Individuals who were sitting for 10 hours or more per day were more likely to be younger, have a high educational level (15 year or more), be inactive in their leisure time smoke, have higher BMI, and had a higher average alcohol consumption level. However, they were less likely to suffer from hypertension (Table 1). Participants not responding to the question on sitting time were more likely to be older (mean = 52), and have less than 12 years of education (41%) but did not vary by other covariates (data not shown).

**Table 1:** **Characteristic of the study population by total sitting time, (n = 71,363)**^**a**^

	**Total sitting time**									
	<6 hours		6-10 hours		>10 hours		p-value	All, complete data^b^		All, Imputed data^c^
Total, n (%)	26,207	(44.6)	23,170	(39.5)	9,327	(15.9)		58,704	(82.3)	
Gender, n (%)										
Men	10,103	(38.5)	9,032	(39.0)	4,404	(47.2)	<0.001	28,181	(39.5)	(39.5)
Women	16,104	(61.5)	14,138	(61.0)	4,923	(52.8)		43,182	(60.5)	(60.5)
Age, mean (SD)	48.8	(14.9)	46.6	(14.9)	44.7	(15.2)	<0.001	48.1	(15.3)	48.1
Education, n (%)										
≤12 years	7,923	(31.2)	5,824	(25.9)	2,237	(24.9)	<0.001	15,984	(28.8)	(30.0)
13-14 years	6,194	(24.4)	5,478	(24.3)	2,289	(25.5)		13,961	(24.5)	(24.4)
≥15 years	11,281	(44.2)	11,227	(49.8)	4,469	(49.7)		26,977	(47.4)	(45.7)
Leisure time physical activity, n (%)										
High/moderate	8,069	(30.9)	6,920	(30.0)	2,415	(26.0)	<0.001	17,404	(29.8)	(28.8)
Low	15,430	(59.2)	12,887	(55.8)	4,635	(49.9)		32,952	(56.3)	(57.0)
Inactive	2,587	(9.9)	3,298	(14.3)	2,246	(24.2)		8,131	(13.9)	(14.2)
Daily smoking, n (%)	3,142	(12.0)	3,059	(13.2)	1,530	(16.4)	<0.001	7,731	(13.2)	(13.8)
Alcohol consumption (drinks/week), mean (SD)	7.5	(8.4)	8.1	(8.8)	8.8	(10.0)	<0.001	8.0	(8.8)	7.9
Body Mass Index (kg/m^2^), mean (SD)	24.8	(4.0)	24.9	(4.2)	25.4	(4.6)	<0.001	24.9	(4.2)	25.0
Hypertension, n (%)	5,009	(19.9)	4,188	(18.7)	1,644	(18.3)	<0.001	10,841	(19.1)	(20.4)
Diabetes, n (%)	714	(2.8)	603	(2.6)	284	(3.1)	0.080	1,983	(2.9)	(2.9)

*Abbreviations*: SD standard deviation, n number of participants.

^a^Subcategories of unweighted numbers in the study population free of coronary heart disease.

^b^The total number of participants varies by covariates due to differences in the number of missing observations. The shown numbers of participants represent complete available information of each covariate.

^c^Multiple imputation techniques used to estimate values for missing data. Shown numbers are proportions (%) for categorical variables and means for continuous variables.

In respect to associations with cardio-metabolic biomarkers, for most variables there was a trend towards an association with poorer outcomes with increasing sitting time (Table 2). Among both men and women, greater daily sitting time was significantly associated with higher levels of cholesterol and triglycerides in the unadjusted associations. However, when adjusted for other covariates the associations were attenuated for women but remained statistically significant for men. The average difference in waist circumference was 2.1 cm for men and 1.6 cm for women between the highest and the lowest category of total sitting time.

**Table 2:** **Cardio-metabolic risk biomarkers according to total sitting time for men and women, (n = 16.049)**^**a**^

		**Cholesterol (mmol/l)**				**Triglycerides (mmol/l)**^**d**^			
	**Total sitting time (hours/day)**	Mean^b^	95% CI	Mean^c^	95% CI	Mean^b^	95% CI	Mean^c^	95% CI
**Men**	< 6	5.21	5.17, 5.24	4.70	4.58, 4.82	1.47	1.44, 1.49	1.17	1.11, 1.25
	6- < 10	5.26	5.18, 5.35	4.73	4.57, 4.90	1.56	1.49, 1.64	1.22	1.12, 1.33
	10+	5.42	5.31, 5.52	4.86	4.67, 5.04	1.64	1.55, 1.73	1.23	1.12, 1.36
*P* for trend		<0.01		<0.01		<0.01		<0.01	
**Women**	< 6	5.34	5.32, 5.48	5.55	5.41, 5.69	1.20	1.18, 1.22	1.34	1.25, 1.43
	6- < 10	5.40	5.33, 5.47	5.59	5.40, 5.78	1.25	1.20, 1.30	1.36	1.24, 1.48
	10+	5.43	5.34, 5.54	5.61	5.40, 5.83	1.30	1.23, 1.37	1.37	1.24, 1.52
*P* for trend		<0.01		0.05		<0.01		0.06	
		**Body mass index (kg/m**^**2**^**)**				**Waist circumference (cm)**			
**Men**	< 6	25.9	25.8, 26.0	25.8	25.4, 26.2	95.4	95.0, 95.7	92.9	91.7, 94.1
	6- < 10	26.0	25.7, 26.3	25.9	25.3, 26.5	96.3	95.4, 97.2	93.8	92.1, 95.5
	10+	26.3	26.0, 26.7	26.2	25.5, 26.8	97.9	96.9, 98.9	95.0	93.1, 96.8
*P* for trend		<0.01		<0.01		<0.01		<0.01	
**Women**	< 6	24.7	24.5, 24.8	24.0	23.4, 24.7	85.5	85.1, 85.8	82.5	80.9, 84.1
	6- < 10	25.0	24.6, 25.3	24.3	23.5, 25.1	86.7	85.8, 87.6	83.5	81.4, 85.6
	10+	25.5	25.0, 26.0	24.6	23.7, 25.5	87.9	86.7, 89.1	84.1	81.7, 86.5
*P* for trend		<0.01		<0.01		<0.01		<0.01	

*Abbreviations*: CI confidence interval, n number of participants.

^a^Includes the study population participating in the clinical health examination. Average values shown are estimated from generalized linear models with imputed values for missing data and weighted by non-response weights.

^b^Apply for a reference person (age 52 years).

^c^Apply for a reference person (age 52 years, alcohol consumption 1-7 drinks per week, never smoker, and education <10 years). Also, analysis with cholesterol and triglycerides applies for a reference person with waist circumference <90 cm and body mass index <25 kg/m2.

^d^Back transformed from the log scale.

Associations of sitting time and MI and CHD incidence as well as all-cause mortality are shown in Table 3. The unadjusted results showed a significantly higher risk for all health outcomes when sitting 10 hours or more per day compared to less than 6 hours per day. Associations were stronger for MI than for CHD and all-cause mortality. No difference was seen for sitting 6-10 hours per day compared to sitting less than 6 hours per day. Although HR’s were lower, the association with myocardial infarction and all-cause mortality remained statistically significant after adjustment for other covariates (HR = 1.38; 95% CI: 1.01, 1.88 and HR = 1.31; 95% CI: 1.09, 1.57, respectively). Although statistically insignificant, the inverse association between sitting time and all-cause mortality persisted after exclusion of participants with a history of cancer and cardiovascular disease (HR = 1.04 [95% CI: 0.85,1.28] and HR = 1.24 [95% CI: 0.97, 1.58] for sitting 6-10 and 10+ hours per day, respectively). Exclusion of events of MI, CHD and death occurring in the first two years of follow-up showed similar results but with wider confidence limits (data not shown). For all-cause mortality, there was evidence of a steady linear increase in mortality rates with each additional hour of total sitting time (HR = 1.03, *P* = 0.01). However, for MI and CHD rates there was no linear increase by total sitting time (MI: HR = 1.00, *P =* 0.79*;* CHD: 1.02, *P = 0.17*).

**Table 3:** **Hazard ratios of Myocardial infarction, coronary heart disease and All-cause mortality by total sitting time, (n = 71,363)**^**a**^

	**Myocardial infarction**						**Coronary heart disease**						**All-cause mortality**					
	Cases (n)	Person, years	HR^b^	95% CI	HR^c^	95% CI	Cases (n)	Person, years	HR^b^	95% CI	HR^c^	95% CI	Cases (n)	Person, years	HR^b^	95% CI	HR^c^	95% CI
**Total sitting time (hours/day)**																		
0- < 6	156	144,137	1.00	Ref	1.00	Ref	674	142,950	1.00	Ref	1.00	Ref	480	145,915	1.00	Ref	1.00	Ref
6- < 10	133	123,427	1.16	0.89, 1.53	1.09	0.83, 1.43	539	122,537	1.01	0.89, 1.15	0.96	0.85, 1.09	397	124,961	1.13	0.97, 1.32	1.05	0.90, 1.22
10+	69	52,367	1.59	1.19, 2.15	1.38	1.01, 1.88	233	50,001	1.19	1.01, 1.40	1.07	0.91, 1.27	197	53,054	1.52	1.27, 1.81	1.31	1.09, 1.57
*P* for trend			<0.01		0.05				0.07		0.59				<0.01		<0.01	

*Abbreviations*: CI confidence interval, HR hazard ratio, n number of participants, Ref Reference category.

^a^The mean follow-up time was 5.4 years. Estimated by Cox regression analyses with imputed values for missing data and weighted by non-response weights. The number of cases reported is the mean of 20 imputed datasets not weighted by non-response weights.

^b^Adjusted for age and sex.

^c^Adjusted for age, sex, education, physical activity level in leisure time, smoking habits, body mass index, alcohol consumption, diabetes, and hypertension.

In particular, adjustment for physical activity in leisure time attenuated the results while adjustment for BMI, diabetes and hypertension made little difference to the results (data not shown). Further adjustment for dietary components did not alter the results or the level of statistical significance (data not shown). When estimating the hazard ratios of CHD and all-cause mortality in individuals with an overall healthy lifestyle, we found no significant association between sitting time and risk of CHD incidence and all-cause mortality. However, there was a tendency to an increased risk for all-cause mortality (HR = 1.44, 95% CI: 0.98, 2.11) (data not shown).

**Figure 1:**
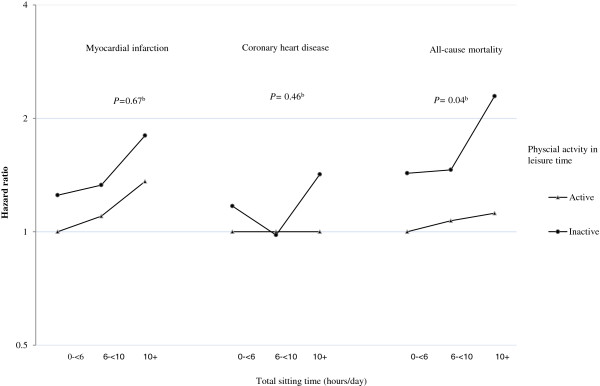
**Hazard Ratios of Myocardial Infarction, Coronary Heart Disease and All-cause Mortality by the Combined Categories of Total Sitting Time and Physical Activity in Leisure Time, (n = 71,363).** The mean follow-up time was 5.4 years. Estimated by Cox regression analyses with imputed values for missing data and weighted by non-response weights. Hazard Ratios are adjusted for age, sex, education, smoking habits, body mass index, alcohol consumption, diabetes, and hypertension. ^b^Total sitting time*Physical activity in leisure time interaction.

Figure 1 shows associations of combined categories of sitting time and physical activity in leisure time and MI and CHD incidence as well as all-cause mortality. Being inactive in leisure time and also sitting more than 10 hours per day in total was associated with increased risk. The multiple adjusted relative risks for the joint effects of high amount of daily total sitting time (10+ hours/day) and physical inactivity in leisure time were 1.80 (95% CI: 1.15, 2.82) for MI, 1.42 (95% CI: 1.11, 1.81) for CHD, and 2.29 (95% CI: 1.82, 2.89) for all-cause mortality, compared with participants who reported sitting the least (<6 hours/day) and who were also physically active in their leisure time (see Additional file 2). Effect modification between leisure time physical activity and sitting time was explored by including an interaction term in the models and this demonstrated a significant interaction for all-cause mortality but not for MI and CHD. Combined analysis with total and leisure-time physical activity measured by IPAQ in combination with sitting time showed fairly similar results (see Additional file 3).

## Discussion

Results from this large prospective population-based study indicate that high amounts of daily sitting time increase the risk of MI incidence and all-cause mortality. Compared to sitting less than 6 hours per day, sitting for 10 hours or more per day was associated with a 38% higher risk of MI and a 31% higher risk of all-cause mortality. For CHD incidence, the picture was less clear. High amounts of daily sitting time combined with physical inactivity in leisure time were associated with increased risk of MI, CHD and all-cause mortality. Being physically active in leisure time attenuated the association between sitting time and all-cause mortality.

To our knowledge, this is the first study on the association between daily total sitting time and CHD incidence in a large cohort of both men and women. Our study reinforces previous work by demonstrating that individuals who sit for 10 hours or more daily were at increased risk of all-cause mortality [8,10-14]. The finding of a significant association between sitting time and MI but not CHD is somewhat ambiguous and in contrast to previous studies showing a positive association between sitting time and cardiovascular mortality [11,12] and between sedentary behavior and CHD incidence [16,18-20]. Comparison with these studies is complicated by differences in the measurements. Sitting while watching television or using a computer are different behavioral settings to other reasons for sitting, and thus may show different risk profiles. Most previous studies of CHD incidence measured sitting time as television viewing time or screen time. Only two studies have examined total sitting time in relation to CHD incidence and these only included women [17,18]. In a large prospective cohort study of 73,743 postmenopausal women, Manson *et al*. found that those who were sitting for 16 or more hours per day had a 68% higher risk for incident CVD compared with less than 4 hours of daily sitting [18]. However, in a recent study of 1654 mid-aged Australian women, Herber-Gast *et al*. found no association between sitting time and cardiovascular disease incidence [17]. Variations in categorization of sitting time may explain some of the differences in the findings as sitting for 16 hours or more is more extreme than a mean of 8.4 hours/day which was considered the highest category in the study by Herber-Gast et al.

This study found that sitting for more than 10 hours per day combined with being physically inactive in leisure time increased the risk of MI and CHD incidence and all-cause mortality. For all-cause mortality, the significant interaction between sitting time and leisure-time physical activity indicated that physical activity in leisure time counterbalanced the risk associated with a higher amount of total daily sitting time. This is in contrast with previous studies documenting the negative effect of sedentary behavior on cardiovascular morbidity and mortality independent of physical activity level [11-13]. However, for MI and CHD, regular leisure time physical activity seemed to attenuate but not eliminate or counterbalance the health consequences of sitting which partly support the independent effect of sitting.

Although, the difference in the cardio-metabolic biomarkers may have little clinical relevance, the indication of a dose response relationship is relevant in understanding potential physiological mechanisms. In both men and women, we found significant associations between increased sitting time and increased levels of the included cardio-metabolic biomarkers. Our finding is in agreement with previous observational studies showing a positive association between sitting time and cardiovascular risk factors including levels of blood lipids [2,27-30].

### Strengths and limitations

The strengths of this study include the prospective population-based design, the large sample of men and women, and the detailed evaluation of participants at baseline, which was conducted by questionnaire in the complete sample and also by clinical health examination in a subsample. Also, an important strength of the study is the use of nation-wide registers to collect data on hospitalizations both prior to baseline and during follow-up. The registers are considered to be of high quality and nearly complete (less than 1% was lost to follow-up) [31,32]. Validation studies have found the national registers to be a valid and powerful tool for estimating the population incidence of MI (predictive values of 82-94% and sensitivity of 78%) whereas, the diagnosis of overall CHD are considered less accurate (predictive value 66%) [33,34]. Presumably, any misclassification of the outcomes was non-differential and will therefore tend to bias the association towards the null because it is not related to the exposure variable (sitting time).

Preclinical or diagnosed disease might influence sitting time and level of physical activity, either biologically or as a stimulant to improve health. If so, this could bias the results. However, individuals with preexisting coronary heart disease were excluded prior to baseline. In addition, also events that occurred during the first 2 years of follow-up and diagnosis of cancers and cardiovascular disease were excluded*.* Although, the associations were attenuated and no longer significant in these sensitivity analyses, they showed the same tendencies.

Although self-reported sitting time is a reasonable well-validated measure, results should be interpreted given the limitations of self-reporting [35]. However, objective measurement instruments were not feasible in a large-scale population study. An advantage of the present study is that it covered the sum of daily sitting during work and in leisure time in both weekdays and weekends. However, the IPAQ sitting question did not include sitting during transportation which also contribute to total sitting time. Therefore, time spent sitting while travelling in a motor vehicle (as likely sitting time) was added, even though the question does not specifically asks for sitting time during transportation. However, we expect the misclassification of sitting time during transportation to be low as standing in a bus or a train often is of short durations, and most travel time is spent sitting.

Physical activity in leisure time was assessed in four predefined categories and further dichotomized in the analyses of the combined effects, which can have resulted in loss of relevant information. Also, the level of physical activity obtained through a questionnaire could be subject to misclassification and likely to have underestimated the associations. Although not validated, the 4-category question on leisure-time physical activity was used for main analyses as it has shown the ability to discriminate between inactive persons and their more active counterparts with respect to maximal oxygen uptake [23] and has shown the ability to predict mortality [36]. The present study focused on leisure-time physical activity only in combination with total sitting time. Physical activity in other domains such as occupation, transport and household contributes to total physical activity but, studies have indicated domain-specific variations in the health benefits of physical activity. In contrast to leisure-time physical activity, the association between occupational physical activity and CHD morbidity and mortality is less clear. To support the findings, results were reanalyzed by applying the IPAQ measure of total and leisure-time physical activity. The IPAQ-questionnaire has been found to be valid measure of total physical activity [22], but as it included a large proportion of missing values (approximately 25%) and no measure of light intensity physical activity, this was used a supplementary measuring instrument. Results were similar although attenuated, which indicate that the applied 4-category question on leisure time physical activity ranks participants according to physical activity similar to the more comprehensive and time consuming IPAQ.

Some factors, such as BMI, hypertension and diabetes, may be regarded as intermediate factors rather than confounding factors and adjusting for these may have conservatively biased our results. Conversely, factors such as triglycerides or total cholesterol were not included as confounding factors as these were regarded as intermediate variables. Confounding from risk factors such as dietary habits, family history of cardiovascular disease and stress were not controlled for which may have influenced results, However, the magnitude of this potential bias is unknown.

Only 14% of the invited individuals participated and thus results may not be representative for the Danish population [21]. To reduce non-response bias, data were weighted for non-response. but given the cohort design of the study, selection should not affect the internal validity. However, relative to other populations, the Danish population is known to be fairly active both in leisure time [37] and in using active transportation, especially bicycling [36]. Thus, it is possible that the results may not apply to a more inactive population.

The relatively short period of follow-up can be viewed both as a strength and a limitation. On one hand, the number of cases is small and provides less statistical power for the analyses. On the other hand, the short period of follow-up is less likely to be influenced by changes in health behaviors over time which may complicate risk estimation.

## Conclusions

Overall, the results of this study suggest that a higher amount of total daily sitting time is associated with greater risk of MI incidence and mortality from all causes. In relation to CHD incidence the results are less clear. Future studies on sitting time and the risk of developing CHD is of great interest to elaborate the inconsistent evidence within this field. In particular, results showed a high risk for participants who were sitting for long hours and also were physically inactive in leisure time. Thus, studies on the interplay between sitting time and physical activity in other domains represent an important area for future research.

## Abbreviations

BMI: Body mass index; CHD: Coronary heart disease; CI: Confidence interval; HR: Hazard ratio; MI: Myocardial infarction; n: Number of participants.

## Competing interests

Financial support: The work was supported by the University of Southern Denmark and the Tryg Foundation. No conflict of interest.

## Authors’ contributions

CBP performed the analysis and wrote the article. AB and JST designed the study and directed its implementation. CBP and JST designed the analytical strategy. AB, MG, JWH and JST supervised the study and assisted the interpretation and implications of the results. LCT assisted in statistical analysis and the implications. All authors certify to have participated sufficiently in the work to take public responsibility for the appropriateness of the design, method, collection, analysis and interpretation of the data. Additionally, all authors have read and approved the final version and have agreed to share data used in the study.

## Supplementary Material

Additional file 1 **Hazard Ratios of Myocardial Infarction, Coronary Heart Disease and All-cause Mortality by Total Sitting Time, (Complete case, n=54,220).**^**a**^ Click here for file

Additional file 2 **Hazard Ratios of Myocardial Infarction, Coronary Heart Disease and All-cause Mortality by the Combined Categories of Total Sitting Time and Physical Activity in Leisure Time, (n=71,363).**^**a**^ Click here for file

Additional file 3 **Hazard Ratios of Myocardial Infarction, Coronary Heart Disease and All-cause Mortality by the Combined Categories of Total Sitting Time and Physical Activity Level Measured by the International Physical Activity Questionnaire (IPAQ), (n=71,363).**^**a**^ Click here for file
