# Supplementary material for: Total sitting time and risk of myocardial infarction, coronary heart disease and all-cause mortality in a prospective cohort of Danish adults
Source: Int J Behav Nutr Phys Act. 2014 Feb 5;11:13. doi: 10.1186/1479-5868-11-13 (PMC3922425; doi:10.1186/1479-5868-11-13)
Supplement: Additional file 1 — Hazard Ratios of Myocardial Infarction, Coronary Heart Disease and All-cause Mortality by Total Sitting Time, (Complete case, n=54,220). a [file 1479-5868-11-13-S1.docx]

Additional file 1. Hazard Ratios of Myocardial Infarction, Coronary Heart Disease and All-cause Mortality by Total Sitting Time, (Complete case, n=54,220).^a^

|  | **Myocardial infarction** | | | | | |  | **Coronary heart disease** | | | | | |  | **All-cause mortality** | | | | | |
| --- | --- | --- | --- | --- | --- | --- | --- | --- | --- | --- | --- | --- | --- | --- | --- | --- | --- | --- | --- | --- |
|  | Cases (n) | Person-years | HR^b^ | 95% CI | HR^c^ | 95% CI |  | Cases (n) | Person-years | HR^b^ | 95% CI | HR^c^ | 95% CI |  | Cases (n) | Person-years | HR^b^ | 95% CI | HR^c^ | 95% CI |
| **Total sitting time (hours/day)** |  |  |  |  |  |  |  |  |  |  |  |  |  |  |  |  |  |  |  |  |
| 0-<6 | 109 | 107,109 | 1.00 | Ref | 1.00 | Ref |  | 461 | 106,382 | 1.00 | Ref | 1.00 | Ref |  | 289 | 108,407 | 1.00 | Ref | 1.00 | Ref |
| 6-<10 | 94 | 95,804 | 1.13 | 0.86, 1.49 | 1.06 | 0.80, 1.40 |  | 387 | 95,198 | 1.07 | 0.93, 1.22 | 1.02 | 0.89, 1.17 |  | 245 | 96,988 | 1.12 | 0.94, 1.33 | 1.05 | 0.88, 1.25 |
| 10+ | 41 | 38,224 | 1.31 | 0.91, 1.88 | 1.13 | 0.78, 1.64 |  | 164 | 37,953 | 1.18 | 0.98, 1.42 | 1.06 | 0.88, 1.28 |  | 119 | 38,702 | 1.39 | 1.12, 1.73 | 1.21 | 0.96, 1.51 |

Abbreviations: CI, confidence interval; HR, hazard ratio; n, number of participants; Ref, Reference category.

^a^ The mean follow-up time was 5.4 years. Estimated by Cox regression analyses and weighted by non-response weights.

^b^ Adjusted for age and sex

^c^ Adjusted for age, sex, education, physical activity level in leisure time, smoking habits, body mass index, alcohol consumption, diabetes, and hypertension
