# Supplementary material for: Total sitting time and risk of myocardial infarction, coronary heart disease and all-cause mortality in a prospective cohort of Danish adults
Source: Int J Behav Nutr Phys Act. 2014 Feb 5;11:13. doi: 10.1186/1479-5868-11-13 (PMC3922425; doi:10.1186/1479-5868-11-13)
Supplement: Additional file 2 — Hazard Ratios of Myocardial Infarction, Coronary Heart Disease and All-cause Mortality by the Combined Categories of Total Sitting Time and Physical Activity in Leisure Time, (n=71,363). a [file 1479-5868-11-13-S2.docx]

Additional file 2. Hazard Ratios of Myocardial Infarction, Coronary Heart Disease and All-cause Mortality by the Combined Categories of Total Sitting Time and Physical Activity in Leisure Time, (n=71,363).^a^

|  |  | **Myocardial infarction** | | | | |  | **Coronary heart disease** | | | | |  | **All-cause mortality** | | | | |
| --- | --- | --- | --- | --- | --- | --- | --- | --- | --- | --- | --- | --- | --- | --- | --- | --- | --- | --- |
| **Leisure time physical activity^b^** | **Total sitting time (hours/day)** | Cases (n) | HR^c^ | 95% CI | HR^d^ | 95% CI |  | Cases (n) | HR^b^ | 95% CI | HR^c^ | 95% CI |  | Cases (n) | HR^b^ | 95% CI | HR^c^ | 95% CI |
| Active | 0-<6 | 130 | 1.00 | Ref | 1.00 | Ref |  | 575 | 1.00 | Ref | 1.00 | Ref |  | 387 | 1.00 | Ref | 1.00 | (Ref) |
|  | 6-<10 | 104 | 1.14 | 0.85, 1.53 | 1.10 | 0.82, 1.48 |  | 443 | 1.02 | 0.88, 1.18 | 1.00 | 0.87, 1.15 |  | 294 | 1.10 | 0.92, 1.32 | 1.07 | (0.89-1.28) |
|  | 10+ | 46 | 1.42 | 0.97, 2.08 | 1.36 | 0.92, 1.99 |  | 156 | 1.05 | 0.85, 1.29 | 1.00 | 0.81, 1.23 |  | 102 | 1.16 | 0.90, 1.48 | 1.12 | (0.87-1.44) |
| Inactive | 0-<6 | 26 | 1.47 | 0.94, 2.29 | 1.25 | 0.80, 1.96 |  | 99 | 1.34 | 1.07, 1.67 | 1.17 | 0.94, 1.46 |  | 94 | 1.53 | 1.19, 1.98 | 1.43 | (1.11-1.84) |
|  | 6-<10 | 29 | 1.66 | 1.08, 2.56 | 1.33 | 0.86, 2.06 |  | 96 | 1.18 | 0.94, 1.49 | 0.98 | 0.78, 1.24 |  | 103 | 1.66 | 1.32, 2.08 | 1.46 | (1.16-1.85) |
|  | 10+ | 23 | 2.38 | 1.55, 3.68 | 1.80 | 1.15, 2.82 |  | 77 | 1.75 | 1.38, 2.23 | 1.42 | 1.11, 1.81 |  | 95 | 2.62 | 2.09, 2.29 | 2.29 | (1.82-2.89) |
| *P* for trend | |  | <0.01 |  | 0.02 |  |  |  | 0.05 |  | 0.37 |  |  |  | <0.01 |  | <0.01 |  |

Abbreviations: CI, confidence interval; HR, hazard ratio; n, number of participants; Ref, reference category.

^a^ The mean follow-up time was 5.4 years. Estimated by Cox regression analyses with imputed values for missing data and weighted by non-response weights. The number of cases reported is the mean of 20 imputed datasets not weighted by non-response weights.

^b^ Physical activity in leisure time was measured as in four-level categories and dichotomized into: ‘Active’ (vigorous, moderate and light activities) and ‘inactive’.

^c^ Adjusted for age and sex

^d^ Adjusted for age, sex, education, smoking habits, body mass index, alcohol consumption, diabetes and hypertension
