# Supplementary material for: Total sitting time and risk of myocardial infarction, coronary heart disease and all-cause mortality in a prospective cohort of Danish adults
Source: Int J Behav Nutr Phys Act. 2014 Feb 5;11:13. doi: 10.1186/1479-5868-11-13 (PMC3922425; doi:10.1186/1479-5868-11-13)
Supplement: Additional file 3 — Hazard Ratios of Myocardial Infarction, Coronary Heart Disease and All-cause Mortality by the Combined Categories of Total Sitting Time and Physical Activity Level Measured by the International Physical Activity Questionnaire (IPAQ), (n=71,363). a [file 1479-5868-11-13-S3.docx]

Additional file 3. Hazard Ratios of Myocardial Infarction, Coronary Heart Disease and All-cause Mortality by the Combined Categories of Total Sitting Time and Physical Activity Level Measured by the International Physical Activity Questionnaire (IPAQ), (n=71,363).^a^

|  | |  |  | **Myocardial infarction** | |  | **Coronary heart disease** | |  | **All-cause mortality** | |
| --- | --- | --- | --- | --- | --- | --- | --- | --- | --- | --- | --- |
| **Physical activity level** | | | **Total sitting time (hours/day)** | HR^b^ | 95% CI |  | HR^b^ | 95% CI |  | HR^b^ | 95% CI |
| **Total physical activity (work, transportation, household and leisure time)** | | |  |  |  |  |  |  |  |  |  |
|  | >2715 MET’s/week | | 0-<6 | 1.00 | Ref |  | 1.00 | Ref |  | 1.00 | Ref |
|  |  | | 6-<10 | 1.09 | 0.81, 1.46 |  | 0.98 | 0.85, 1.14 |  | 1.03 | 0.87, 1.23 |
|  |  | | 10+ | 1.32 | 0.88, 1.98 |  | 1.13 | 0.93, 1.38 |  | 1.11 | 0.85, 1.45 |
|  | 0-2715 MET’s/week | | 0-<6 | 0.96 | 0.62, 1.48 |  | 1.17 | 0.95, 1.44 |  | 1.19 | 0.92, 1.54 |
|  |  | | 6-<10 | 1.18 | 0.77, 1.79 |  | 1.03 | 0.84, 1.26 |  | 1.36 | 1.10, 1.68 |
|  |  | | 10+ | 1.61 | 1.05, 2.48 |  | 1.20 | 0.93, 1.54 |  | 1.92 | 1.52, 2.43 |
| **Leisure time physical activity** | | |  |  |  |  |  |  |  |  |  |
|  | >318 MET’s/week | | 0-<6 | 1.00 | Ref |  | 1.00 | Ref |  | 1.00 | Ref |
|  |  | | 6-<10 | 1.27 | 0.91, 1.65 |  | 1.00 | 0.86, 1.15 |  | 1.08 | 0.90, 1.28 |
|  |  | | 10+ | 1.29 | 0.87, 1.92 |  | 1.07 | 0.88, 1.31 |  | 1.18 | 0.93, 1.49 |
|  | 0-318 MET’s/week | | 0-<6 | 1.29 | 0.89, 1.88 |  | 1.15 | 0.95, 1.40 |  | 1.13 | 0.88, 1.42 |
|  |  | | 6-<10 | 1.12 | 0.72, 1.74 |  | 1.01 | 0.81, 1.25 |  | 1.22 | 0.97, 1.53 |
|  |  | | 10+ | 2.06 | 1.33, 3.18 |  | 1.35 | 1.07, 1.73 |  | 1.91 | 1.48, 2.46 |

Abbreviations: CI, confidence interval; HR, hazard ratio; n, number of participants; Ref, reference category.

^a^ The mean follow-up time was 5.4 years. Estimated by Cox regression analyses with imputed values for missing data and weighted by non-response weights

^b^ Adjusted for age, sex, education, smoking habits, body mass index, alcohol consumption, diabetes and hypertension
